# Silibinin, A Natural Blend In Polytherapy Formulation For Targeting Cd44v6 Expressing Colon Cancer Stem Cells

**DOI:** 10.1038/s41598-018-35069-0

**Published:** 2018-11-19

**Authors:** Shanaya Patel, Bhargav Waghela, Kanisha Shah, Foram Vaidya, Sheefa Mirza, Saumya Patel, Chandramani Pathak, Rakesh Rawal

**Affiliations:** 1 Division of Biological & Life Sciences, School of Arts and Sciences, Ahmedabad University, Ahmedabad, Gujarat India; 2 Department of Life Sciences, School of Sciences, Gujarat University, Ahmedabad, Gujarat India; 3 Department of Cell Biology, Indian Institute of Advanced Research, Gandhinagar, Gujarat India

## Abstract

Colon cancer stem cells have been attributed to poor prognosis, therapeutic resistance and aggressive nature of the malignancy. Recent reports associated CD44v6 expression with relapse, metastasis and reduced 5-year survival of colon cancer patients, thereby making it a potential therapeutic target. Thus, in this study, comprehensive prediction and screening of CD44v6 against 1674 lead compounds was conducted. Silibinin was identified as a potential compound targeting CD44v6. Inorder to substantiate these findings, the cytotoxic effect of 5FU, Silibinin and 5FU+ Silibinin was assessed on human colon carcinoma cell line HCT116 derived CD44+ subpopulation. 5FU+ Silibinin inhibited cell proliferation of CD44+ subpopulation at lower concentration than Silibinin standalone. Further, corresponding to CD44v6 knockdown cells, 5FU+ Silibinin treatment significantly decreased CD44v6, Nanog, CTNNB1 and CDKN2A expression whereas increased E-cadherin expression in HCT116 derived CD44+ cells. Moreover, synergistic effect of these drugs suppressed sphere formation, inhibited cell migration, triggered PARP cleavage and perturbation in mitochondrial membrane potential, thereby activating intrinsic apoptotic pathways and induced autophagic cell death. Importantly, 5FU+ Silibinin could inhibit PI3K/MAPK dual activation and arrest the cell cycle at G0/G1 phase. Thus, our study suggests that inhibition of CD44v6 attenuates stemness of colon cancer stem cells and holds a prospect of potent therapeutic target.

## Introduction

Colon cancer is one of the most commonly diagnosed malignancies worldwide with a radically increased rate of morbidity and mortality as compared to other malignancies^1,2^. Currently, in addition to surgery, 5-fluorouracil (5-FU) in combination with other anti-cancer agents is used as the standard first line chemotherapy based on NCCN guidelines^1^. Despite of these advancements in the therapeutic regimen, several studies attribute failures of the conventional chemotherapy to a distinct subpopulation of quiescent cancer cells referred to as “Cancer Stem Cells”. The cancer stem cell (CSC) hypothesis is rising to be an attractive cellular mechanism that proposes a hierarchical organization within the tumor bulk and justifies the functional heterogeneity of solid tumors responsible for the aggressive nature of the malignancy and therapeutic refractoriness^3–5^.

CD44, a widely expressed membrane adhesion molecule, is reported to be responsible for various biological and functional processes such as cell adhesion, growth, epithelial-mesenchymal transition (EMT) and tumor progression^6,7^. CD44 transcripts undergo complex alternative splicing, resulting in functionally different isoforms expressed primarily on epithelial cells^8^. Although the expression of standard isoform (CD44s) has been more extensively studied, the variant isoforms (CD44v) are reported to have an indispensable role in cancer progression and development^8,9^. Amongst these isoforms, CD44v6 has been characterized as a functional marker which has been associated with tumor growth, metastasis, recurrence, poor prognosis and reduced 5-year survival of colon cancer patients, thereby indicating the imperative significance of this CSC marker as an effective therapeutic target^9–11^. Therefore, the need of the hour is to identify potential lead compounds that facilitate in development of anti-CD44v6 therapeutic modalities, assess the efficacy of these drugs on molecular and functional mechanisms of CD44v6 and evaluate their ability to target the pathways regulating this subpopulation. Targeting this tumor initiating cell population would have a significant impact in improving the 5-year survival rate by decreasing incidence of therapeutic resistance, relapse and metastasis in colon cancer patients^12–14^.

In spite of the impending therapeutic significance of CD44v6, absence of a comprehensively modelled structure of this protein hampers the process of identification and development of potential lead compounds. Thus, this study aims to predict human CD44v6 protein structure, screen various lead compounds against CD44v6 and identify a potential lead compound by homology modeling, molecular docking and molecular dynamic simulation approach. Furthermore, we sought to investigate the role of identified potential drug compounds on cancer stem-like CD44+ cells from the human colon carcinoma cell line HCT116 in order to explore the impact of drug based suppression of CD44v6 on molecular and functional characteristics such as anchorage independent growth potential, migration, expression of vital stemness and EMT markers, cell cycle regulation, induction of apoptotic and autophagic mechanisms and various downstream signaling pathways. An in-depth analysis of CD44v6 with these compounds would thereby provide newer avenues for development of CSC-targeted therapies in future.

## Results

### Protein structure prediction and lead compound identification for CD44v6

**Figure 1:**
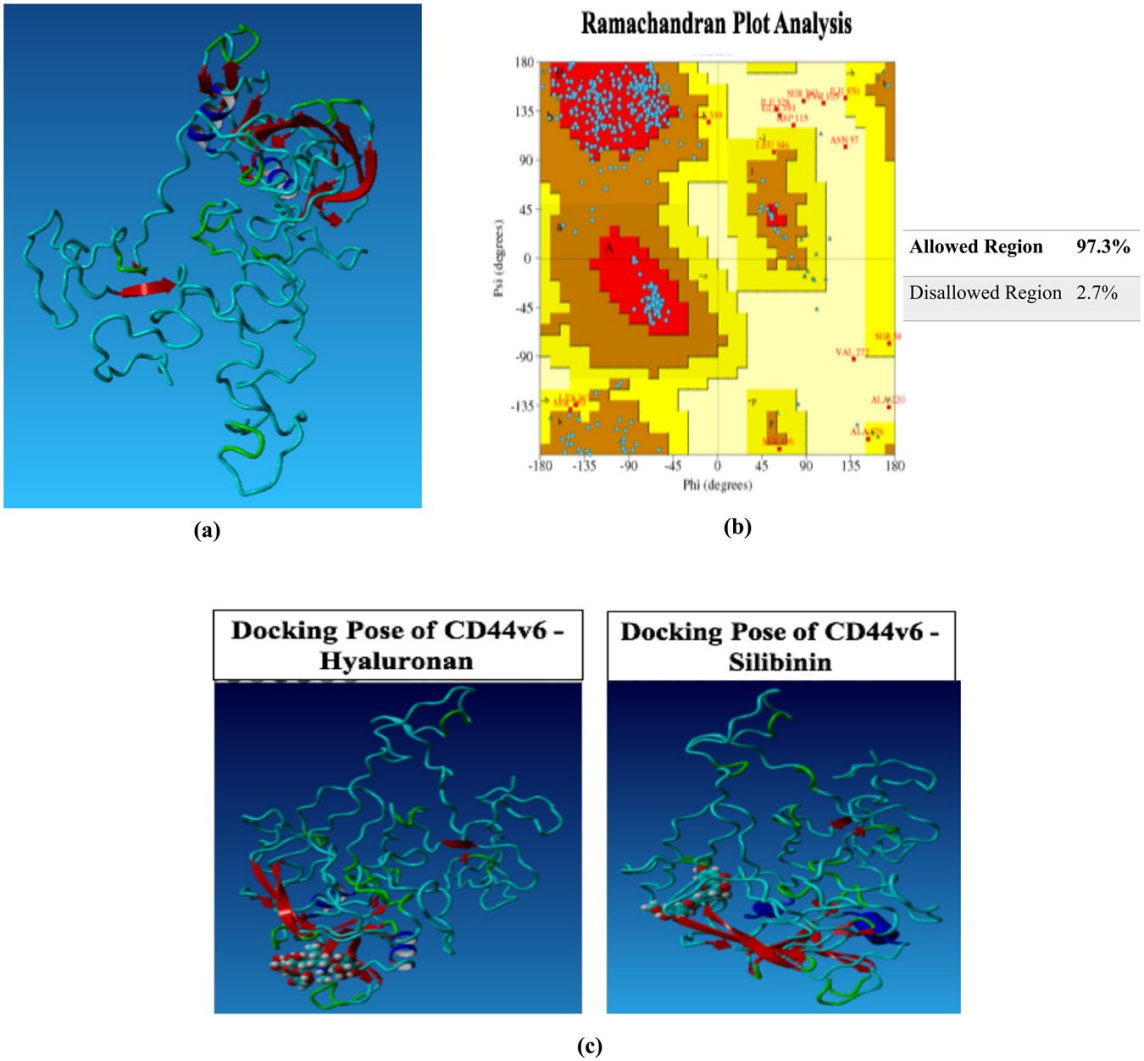
Structure prediction of CD44v6 and virtual screening of potential lead compounds targeting CD44v6. (**a**) Comprehensive prediction of three-dimensional CD44v6 protein structure using template based homology modeling. (**b**) Ramachandran plot analysis assessing the sensitivity and specificity of the predicted structure. (**c**) 3D representation of the complex between Hyaluronan (HA) and Silibinin in the HA-binding domain of CD44v6 by virtual molecular docking.

Three-dimensional model of CD44v6 protein structure was predicted using template-based homology modeling approach. 1UUH, 2PF5, 4DUR and 4MRH (PDB structures) were identified as suitable templates for modeling as they demonstrated high sequence similarity with CD44v6 sequence. Further, Ramachandran plot analysis demonstrated presence of 97.30% of all residues in the allowed regions, thereby substantiating the accuracy of this predicted structure (Fig. 1a,b). Thus, FDA approved drugs, experimental drugs and natural compounds were screened against this modelled structure of CD44v6 in order to identify potential lead compound on the basis of its binding energy, binding pattern and dissociation constant score. The docking results depicted that Silibinin bound to CD44v6 with a significantly higher binding affinity (7.23 kcal mol^−1^) as compared to hyaluronan in its own binding pocket (6.23 kcal mol^−1^; Table 1; Fig. 1c). Moreover, difference in the interacting residues and H-bonds of CD44v6 with HA and Silibinin were analyzed. These results demonstrated that HA and Silibinin interacted with 20 and 16 contacting amino acid residues of CD44v6 respectively. Importantly, Silibinin interacted with CD44v6 in the same binding pocket as HA but did not interact with any common amino acid residues. Subsequently, the H-bond interaction analysis depicted that HA interacted with Ile26, Thr27, Gly40, Tyr42, Glu75 and Cys77 amino acids, whereas Silibinin interacted with amino acid residues Ala9, Trp10, Tyr161, Leu327 of CD44v6 via H-bonds (Table 1). Thus, based on the binding energy, contacting residues and H-bond interactions within HA binding pocket, we identified Silibinin as a potential lead compound that could competitively inhibit HA from binding with CD44v6.

**Table 1:** Binding energy and bonding interactions formed by Salinomycin, Silibinin and HA within the binding pocket of CD44v6.

Compounds	Binding Energy	H-bond Interactions	Steric Interactions
HA	6.23	Ile26, Thr27, Gly40, Tyr42, Glu75, Cys77	Ile26, Thr27, Glu37, Gly40, tyr42, Glu75, Cys77, Arg78, Arg150
Silibinin	7.23	Ala9, Trp10, Tyr161, Leu327	Trp247, His248, Arg252, Lys256, Ser380, Thr393, Asn395

### Insight into structural perturbation and interaction profile of CD44v6 bound with HA and Silibinin

**Figure 2:**
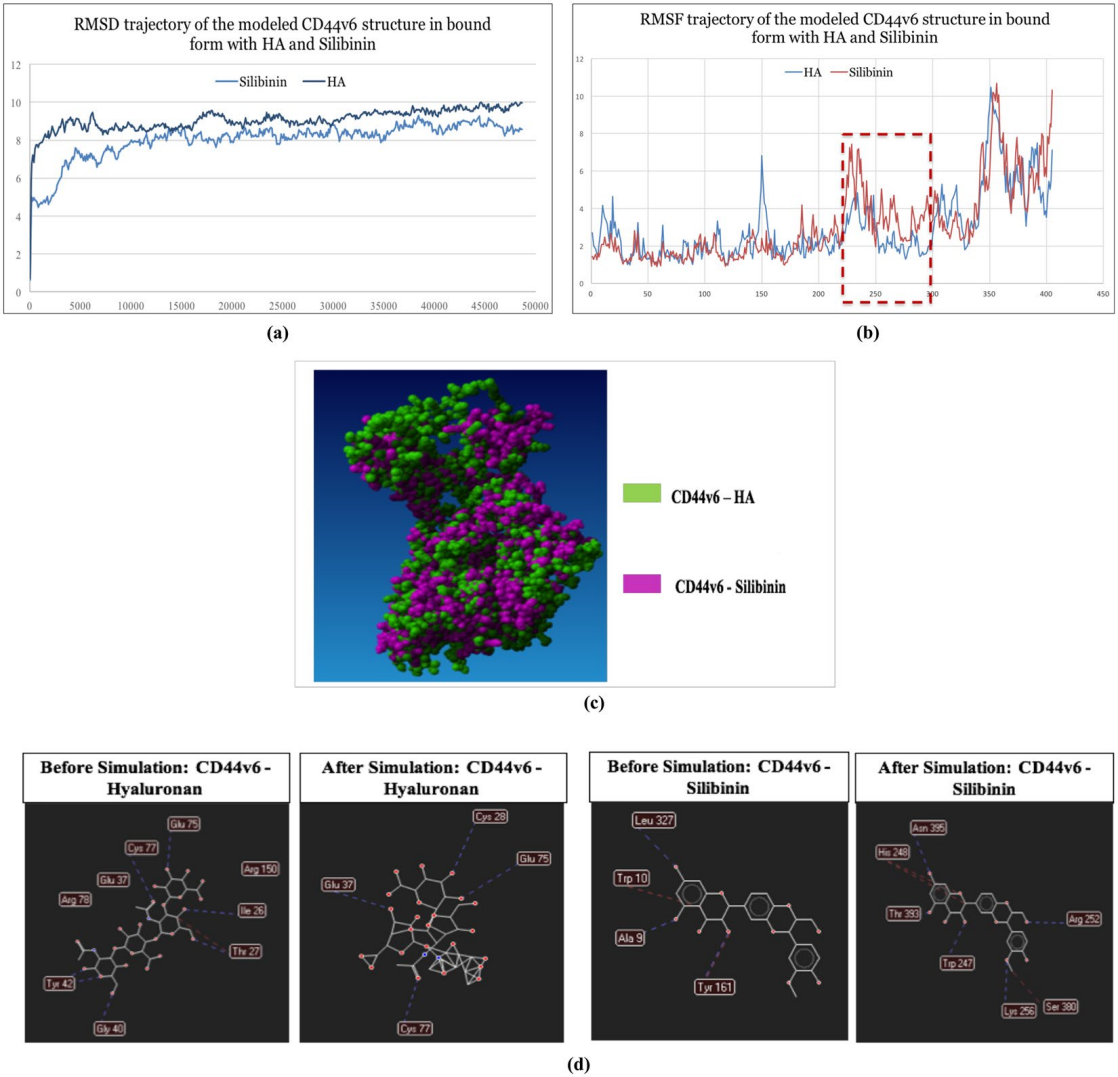
Molecular dynamics, molecular modeling and structural perturbation analysis of CD44v6 by selected potential drug compound. (**a**) RMSD trajectory analysis of the modeled CD44v6 structure in bound form with HA and Silibinin during 50 ns long MD simulations. Notes: Duration of MD simulations scaled on X-axis and Y-axis on the left side represents the RMSD deviation of protein structure in Å. Y-axis on the right side represents the HA ligand RMSD trajectory in their respective binding pockets. (**b**) RMSF analysis of the modeled CD44v6 structure in bound form with HA and Silibinin during 50 ns long MD simulations. The highlighted regions (red) is depicting ligand induced internal residual fluctuations in CD44v6 structure captured during MD simulation process. (**c**) 3D representation of the superimposed complexes of CD44v6 bound to HA (green) and Silibinin (pink) respectively demonstrating ligand induced conformational change in the structure of CD44v6 protein. (**d**) Difference in H-bond interaction profile of CD44v6 with HA and Silibinin before and after MD simulation process.

In order to analyze the ligand induced dynamic stability and local structural changes of CD44v6 structure, RMSD and RMSF changes of C-α atoms of CD44v6-Silibinin and CD44v6-HA complexes were plotted against time (50 ns)^15^. CD44v6-Silibinin complex depicted relatively lower average RMSD (8.00 Å) compared to CD44v6–HA complex (9.05 Å), indicating a higher stability of Silibinin with CD44v6 as compared to its natural ligand (Fig. 2a). Moreover, CD44v6-HA complex showed higher overall per-residue fluctuations as compared to CD44v6-Silibinin complex, prompting towards binding mediated internal conformational instability. Interestingly, higher fluctuations were observed in amino acid residues from 224–295 of the CD44v6-Silibinin complex which not only comprises of the v6 variant insertion but also subsequent transmembrane region (Fig. 2b). On super-imposing the post simulation-derived bound structures of CD44v6-Silibinin and CD44v6-HA complexes, a significant ligand binding-induced global conformational change was observed (Fig. 2c). Collectively, these findings substantiated that Silibinin might have potential role in targeting CD44v6 as it has a significant effect in modulating the conformation of functionally relevant amino acids while maintaining the overall stability of CD44v6 protein.

Furthermore, difference in hydrogen bonds and hydrophobic interaction profiles of Silibinin with CD44v6 as compared to HA were assessed throughout the 50 ns simulation. CD44v6 interacted with HA via 15 bonding interactions amongst which 6 were H-bond interaction. A loss of 4 H-bonds was observed while Cys28 and Glu37 amino acid residues made new H-bond interactions with HA during the simulation process (Fig. 2d). Interestingly, CD44v6 and Silibinin showed limited bonding interactions as compared to HA. Amongst these, CD44v6 interacted with Silibinin via 4 H-bond interactions; however, post simulation of Silibinin demonstrated a loss of all 4 H-bond contacts and made 7 newer H-bond interactions that sustained throughout 50 ns of simulation process (Fig. 2d). Thus, based on these results it could be hypothesized that CD44v6-Silibinin interactions might be responsible for competitively inhibiting HA from binding with CD44v6 and stably holding Silibinin in the HA binding pocket.

### Silibinin alone and in synergism with conventional chemotherapeutic agents induced cytotoxic effect on HCT116 derived CD44+ cancer stem like cell population

**Figure 3:**
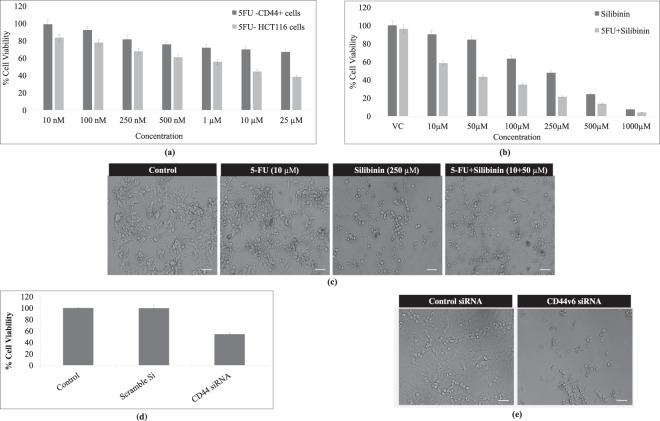
Cytotoxic effect of 5FU, Silibinin and Silibinin+ 5FU on cell proliferation of HCT116 derived CD44+ subpopulation. (**a**) Determination of cytotoxic effects of 5FU on HCT116 cell line and HCT116 derived CD44+ subpopulation. The graph represents the percentage of cell viability at various concentrations (10 nM, 100 nM, 250 nM, 500 nM, 1 μM, 10 μM, 25 μM) of 5FU for 48 h. (**b**) Assessment of cytotoxic effects of Silibinin and Silibinin+ 5-FU on cell viability of HCT116 derived CD44+ subpopulation at various concentrations (10 μM, 50 μM, 100 μM, 250 μM, 500 μM, 1000 μM) of silibinin standalone and in synergism with 10 μM of 5FU for 48 h. (**c**) Morphological analysis of 5FU, Silibinin and Silibinin+ 5FU treated CD44+ cells by Bright–field microscopy. Scale bar:10 μm. (**d**) Evaluation of the inhibitory effects of CD44v6 siRNA (10 nm) on HCT116 derived CD44+ cells as compared to control for 48 h. (**e**) Morphological analysis of CD44v6 knockdown cells by Bright–field microscopy as compared to scramble control (Control siRNA). Scale bar:10 μm. Note: DMSO was used as a vehicle control. Error bars represent mean ± SEM of three independent experiments with p-value indicated as *p < 0.05, **p < 0.01 and ***p < 0.001.

On the basis of the above findings, it became indispensable to assess the cytotoxic potential of Silibinin stand alone or in synergism with the conventional chemotherapeutic agent on HCT116-CD44+ enriched subpopulation. The anti-proliferative ability of this compound was assessed in comparison to 5-Florouracil (5-FU) and CD44v6 specific siRNA transfected cells which were considered as experimental controls in order to validate the sensitivity and specificity of all the molecular and functional assays. 5FU standalone demonstrated a significant cytotoxic effect on HCT116 cell line at its IC_50_ (10 μM) after 48 h of treatment; however, it failed to demonstrate similar cytotoxic effect on HCT116 derived CD44+ cells at its IC_50_ or even at higher concentrations (Fig. 3a). The IC_50_ of Silibinin and 5FU+ Silibinin combination (10–1000 μM each) were determined from the dose-response curve after 48 h of treatment on CD44+ subpopulation derived from HCT116 cells. Based on the cytotoxicity assay, 5FU+ Silibinin (10 μM + 50 μM respectively) significantly decreased the percentage of CD44+ cells to 43.34 ± 2.16% as compared to 47.83 ± 2.39% in Silibinin (250 μM) treated HCT116-CD44+ cells respectively (Fig. 3b,c). However, 5FU+ Silibinin demonstrated significant increase in the growth inhibition rate in CD44v6 siRNA transfected HCT116-CD44+ compared to the scramble control (p < 0.01; Fig. 3d,e). These results indicate that 5FU in synergism with Silibinin effectively inhibited cell proliferation of CD44+ subpopulation of HCT 116 cells at lower concentrations. Thus, 5FU-Silibinin combination at the given concentration was selected for further assessment of various molecular and functional assays.

### Silibinin in combination with 5FU significantly downregulated CD44v6 expression and altered the gene expression of vital stemness and EMT markers in HCT116 cell line

**Figure 4:**
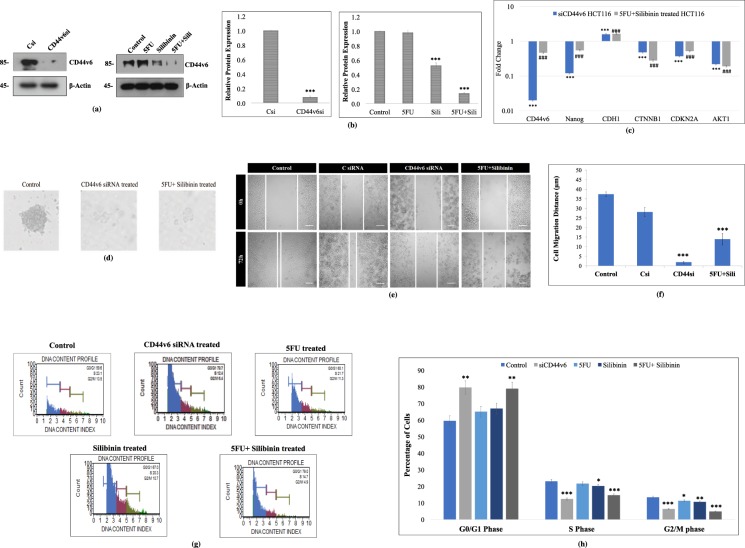
Effect of 5FU, Silibinin and 5FU+ Silibinin on gene and protein expression profile, sphere forming ability, migratory potential and cell cycle mechanism of HCT116 derived CD44+ subpopulation. (**a**) Western Blot analysis of CD44v6 in HCT116 derived CD44+ cells upon treatment with CD44v6 siRNA (10 nM), 5FU (10 μM), Silibinin (250 μM) and combinatorial treatment of 5FU+ Silibinin (10 + 50 μM). β-actin was used as a loading control. (**b**) Densitometric analysis of western blot bands of CD44v6 protein post treatment with CD44v6 siRNA, 5FU, Silibinin and 5FU+ Silibinin. Error bars represent mean ± SEM of three independent experiments with p-value indicated as *p < 0.05, **p < 0.01 and ***p < 0.001. (**c**) mRNA expression of various markers was analyzed by qRT-PCR. The expression of mRNA of the respective genes (CD44v6, Nanog, CDH1, CTNNB1, CDKN2A and Akt1) was evaluated in CD44v6 siRNA (10 nM) and 5FU+ Silibinin (10 + 50 μM) treated cells as compared to their corresponding control. The data was normalized with 18 s rRNA (endogenous control). Error bars represent mean ± SEM of three independent experiments with p-value indicated as ***p < 0.001 for CD44v6 siRNA transfected cells and ^###^p < 0.001 for 5FU+ Silibinin treated cells normalized against their respective controls. (**d**) Sphere forming ability of HCT116 derived CD44+ subpopulation was analyzed post CD44v6 specific siRNA and 5FU+ Silibinin treatment (100x magnification). The data shown are representative of three isolated experiments. (**e**) The migration ability of HCT116 derived CD44+ subpopulation was monitored in CD44v6 knockdown and 5FU+ Silibinin treated condition by wound healing assay. DMSO was used as a vehicle control. The data shown are representative of three isolated experiments. (**f**) The graphs represent the relative distance migrated by cells per field (μm) in the wound healing assay. Error bars represent mean ± SEM of three independent experiments with p-value indicated as *p < 0.05, **p < 0.01 and ***p < 0.001. (**g**) Alteration in cell cycle mechanism was assessed using Muse^TM^ analyser based flow cytometric analysis. (**h**) The graph indicates the percentage of cells in each phase of the cell cycle. Error bars represent mean ± SEM of three independent experiments with p-value indicated as *p < 0.05, **p < 0.01 and ***p < 0.001.

In order to validate the effect of 5FU, Silibinin and 5FU+ Silibinin in targeting CD44v6, we evaluated protein expression of this CD44 isoform as compared to control cells post 48 h. CD44v6 siRNA transfected cells (post 48 h) were considered as experimental controls. Western blot results indicated that both Silibinin standalone and 5FU+ Silibinin combinatorial treatment significantly inhibited the protein expression of CD44v6 in HCT116 derived CD44+ cells; however, 5FU+ Silibinin suppressed CD44v6 expression with greater efficacy (at a relatively lower concentration) than Silibinin alone and at similar effectiveness as siRNA mediated transient silencing (Fig. 4a,b). Based on these results, 5FU+ Silibinin treated and CD44v6 knockdown cells were further subjected to gene expression analysis post 48 h of treatment. Moreover, Real time PCR analysis showed that CD44v6 mRNA expression was significantly decreased in both CD44+ knockdown cells as well as Silibinin+ 5FU treated cells as compared to the control (p < 0.01) (Fig. 4c). Intriguingly, both CD44v6 siRNA transfected and Silibinin+ 5FU treated cells demonstrated a significant decrease in the mRNA expression patterns of Nanog, AKT1, CTNNB1 and CDKN2A and a notable increase in the expression levels of CDH1 (Fig. 4c). Thus, our findings suggest that combinatorial effect of 5FU+ Silibinin has a significant effect on the expression of CD44v6 and other markers that have an inevitable role in modulating processes like EMT, cell cycle mechanism and various signalling pathways.

### Synergistic effect of Silibinin and 5-Fluorouracil effectively suppresses anchorage independent growth and migratory potential of HCT116 derived CD44+ cells

Since self-renewal and aberrant differentiation are characteristic features of cancer stem cells, it became imperative to assess the synergistic effect of Silibinin+ 5FU on the clonogenic ability and migratory potential of HCT116 derived CD44+ subpopulation. To examine the synergistic effect of Silibinin+ 5FU on clonogenic properties of HCT116-CD44+ subpopulation as compared to the control or CD44v6 specific siRNA transfected cells. We examined the number of spheres generated in the absence or presence of Silibinin+ 5FU post 48 h of treatment. The colony forming assay demonstrated that Silibinin+ 5FU treated HCT116-CD44+ cells significantly decreased both the mean number and size of the colonospheres and inhibited the sphere forming ability of CD44+ cells. Interestingly, the effects of Silibinin+ 5FU depicted nearly similar efficacy and results on sphere formation as observed in siCD44v6 treated CD44+ cells (Fig. 4d). Collectively, Silibinin+ 5FU mediated reduction in the number and size of tumor spheres signifies that synergistic effect of this drug combination not only inhibits the cancer stem cell population but also significantly reduces the bulk tumor cells.

Further, it has been reported that CD44 subpopulation has the ability to promote cell migration through the degradation of ECM components by metalloproteinases^16,17^. Therefore, we examined the effects of Silibinin+ 5FU on the migratory potential of HCT116-CD44+ cells. The cell migration kinetic analysis revealed that synergistic effect of Silibinin+ 5FU significantly reduced the migration of HCT116 derived CD44+ cells in a time- and dose-dependent manner as compared to the DMSO treated control cells after 72 h. Significant reduction was observed in the cell migration distance and number of migrated cells as early as 24 h in the treated cells (Fig. 4e,f). Interestingly, inhibition of cell migration was more prominent in Silibinin+ 5FU combination treated cells as compared to the CD44v6 siRNA transfected cells (Fig. 4e,f). Thus, these results implied that synergistic effect of Silibinin+ 5FU on CD44+ cells effectively suppressed the self-renewal and inhibited the migratory potential of HCT116-CD44+ cells. However, self-renewal and differentiation of CSCs is greatly interconnected with the cell cycle mechanism, hence we further sought to assess the synergistic effect of Silibinin+ 5FU on cell cycle mechanism of CD44+ subpopulation.

### Silibinin and 5FU combinatorial treatment on HCT116-CD44+ cells cause G0/G1 phase arrest of the cell cycle mechanism

The conventional chemotherapeutic agents are known to modulate the growth capability of cancer cells by specifically targeting particular cell cycle phases. HCT116-CD44+ cells treated with 5FU, Silibinin and combination of Silibinin+ 5FU were subjected to cell cycle analysis in order to assess the number of cells in each phase of the cell cycle. The results of cell cycle analyses indicated that siRNA mediated silencing of CD44v6 resulted in G0/G1 arrest (p < 0.0031) with simultaneous decrease in the S (p < 0.015) and G2/M (p < 0.043) phases as compared to the control (Fig. 4g,h). Similar to these results, the synergistic effect of 5FU and Silibinin also demonstrated 19.4% (p < 0.0025) increase in accumulation of cells in G0/G1 phase as compared to control. Contradictory to these findings, 5FU and Silibinin standalone treated cells failed to demonstrate a significant arrest in the G0/G1 phase of the cell cycle as compared to their corresponding control (Fig. 4g,h). Additionally, siRNA knockdown and 5FU+ Silibinin treated cells demonstrated a significant reduction in S (p < 0.0001 and p < 0.0004 respectively) and G2/M (p < 0.0002 and p < 0.0001 respectively) phases of the cell cycle respectively. Moreover, Silibinin treated CD44+ cells demonstrated a comparative reduction in S (p < 0.033) and G2/M (p < 0.004) phase; however, this decrease was not as substantial as demonstrated by the synergistically treated cells (Fig. 4g,h). Thus, we further sought to assess the underlying mechanism by which 5FU+ Silibinin combination increased the sensitivity of CSC population to apoptosis.

### Down regulation of CD44v6 and treatment of 5-FU+ Silibinin induces apoptotic cell death in HCT-116 cells

**Figure 5:**
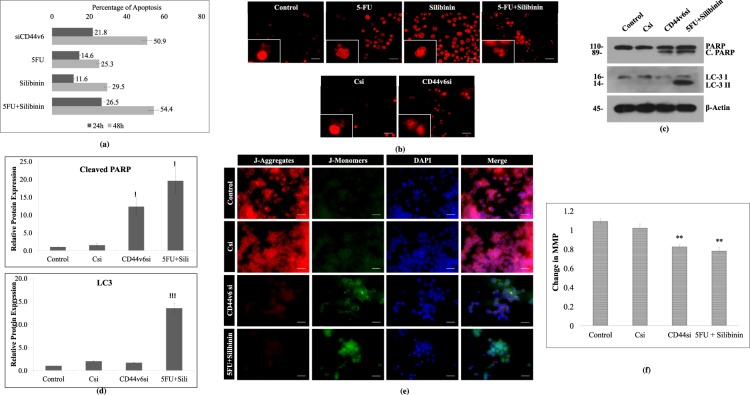
Enhanced effect of 5FU+ Silibinin combinatorial treatment on apoptotic mechanism of CD44+ subpopulation. (**a**) Apoptotic cell death analysis was conducted by Annexin-V/PI staining of CD44v6 siRNA (10 nM), 5FU (10 μM), Silibinin (250 μM) and 5FU+ Silibinin (10 + 50 μM) treated cells as compared to untreated cells using the Muse^TM^ analyser. The graph indicates the percentage of apoptotic and live cells in treated cells compared to untreated cell population. Error bars represent mean ± SEM of three independent experiments with p-value indicated as *p < 0.05, **p < 0.01 and ***p < 0.001. (**b**) Nuclear morphology and fragmentation of CD44v6 siRNA (10 nM), 5FU (10 μM), Silibinin (250 μM) and 5FU+ Silibinin (10 + 50 μM) treated cells were stained with PI and observed under fluorescent microscope. Scale bar:10 μm. (**c**) Western Blot analysis of PARP cleavage and LC-3 I/II conversion in HCT116 derived CD44+ cells upon CD44v6 knockdown (10 nM) and combinatorial treatment of 5FU+ Silibinin (10 + 50 μM). β-actin was used as a loading control. (**d**) Densitometric analysis of western blot bands of PARP cleavage and LC-3 I/II conversion post treatment with CD44v6 siRNA and 5FU+ Silibinin compared to their control counterparts. Error bars represent mean ± SEM of three independent experiments with p-value indicated as *p < 0.05, **p < 0.01 and ***p < 0.001. (**e**) Analysis of changes in mitochondrial membrane potential (MMP) via JC-1 dye by fluorescence microscopy. Scale bar: 10 μm. (**f**) Quantitative analysis of loss of mitochondrial membrane potential (Ψ). Error bars represent mean ± SEM of three independent experiments with p-value indicated as *p < 0.05, **p < 0.01 and ***p < 0.001.

To elucidate the effect of 5FU, Silibinin and 5FU+ Silibinin on apoptotic cell death, we performed annexin-V/PI staining at 24 h and 48 h time intervals post treatment using Muse Cell Analyzer. The results revealed that 5FU+ Silibinin demonstrated 54.4% apoptotic cell death at 48 h which was analogous to CD44v6 siRNA transfected cells (50.9%), thereby suggesting that down regulation of CD44v6 induces apoptosis in HCT-116 derived CD44+ cell subpopulation. However, more promising and significant apoptotic cell death was observed upon subjecting these cells to combinatorial treatment of 5FU+ Silibinin as compared to Silibinin and 5FU standalone treatment (Fig. 5a). Moreover, the propidium iodide nuclear staining also showed significant number of PI positive cells with prominent characteristics of apoptosis such as nuclear condensation and fragmentation in CD44v6 siRNA and Silibinin+ 5FU treated cells as compared to individual treatment of 5FU and Silibinin (Fig. 5b). Based on these findings, it was crucial to understand the underlying mechanism by which combinatorial drug treatment pushed the CD44+ subpopulation towards apoptotic cell death. Thus, PARP cleavage, LC3 conversion and mitochondrial membrane potential analysis of 5FU+ Silibinin and siRNA knockdown cells was conducted. Western blot analysis clearly depicted cleavage of PARP protein in both siCD44v6 transfected and Silibinin+ 5FU treated cells, thereby indicating apoptosis mediated cell death in these cells (Fig. 5c,d).

Since reduction in mitochondrial membrane potential is a key event during apoptotic cell death, we monitored the mitochondrial membrane potential both qualitatively and quantitatively using JC-1 staining. Our analysis demonstrated a significant reduction of mitochondrial membrane potential in CD44v6 siRNA transfected and 5FU+ Silibinin treated cells (Fig. 5e,f). Moreover, we analyzed the effect of siRNA mediated silencing of CD44v6 and co-treatment of Silibinin+ 5FU on autophagy mechanism by western blotting of LC-3 I/II. Our results have shown that co-treatment of 5FU and silibinin induces LC-3 I/II conversion while down regulation of CD44v6 have not shown LC-3 I/II conversion (Fig. 5c,d). Thus, these findings clearly depicted that synergistic effect of Silibinin and 5FU had the ability to induce autophagic cell death which was not observed in CD44v6 silenced cells.

### Silibinin in synergism with 5FU leads to inactivation of PI3K/AKT-MAPK dual signaling pathways of CD44+ HCT116 cells

**Figure 6:**
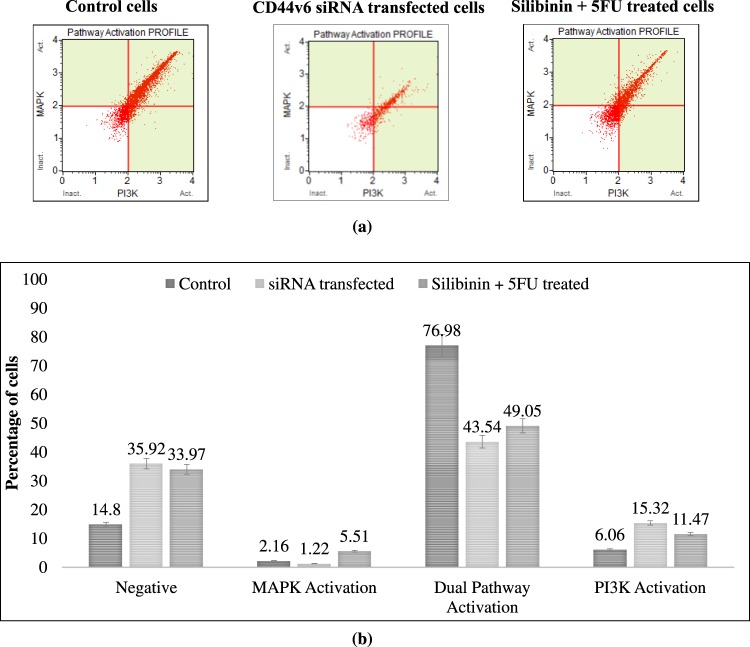
Synergistic effect of 5FU and Silibinin on activation of PI3K/MAPK dual pathway in CD44 subpopulation. The effect of silencing and combinatorial drug treatment induced CD44v6 suppression was examined on PI3K/MAPK dual pathway activation of HCT116 derived CD44+ cells. (**a**) PI3K/MAPK dual activation analysis was conducted on CD44v6 siRNA (10 nM) and 5FU+ Silibinin (10 + 50 μM) treated cells as compared to their untreated counterparts using the Muse^TM^ analyser based flow cytometry. (**b**) The graph indicating the percentage of cells in treated cells compared to untreated cell population. Error bars represent mean ± SEM of three independent experiments with p-value indicated as *p < 0.05, **p < 0.01 and ***p < 0.001.

CSCs trigger certain signaling pathways and direct cross-signaling between various signaling pathways which eventually modulate various processes such as self-renewal, tumorigenesis, cell proliferation, migration, apoptosis and cell cycle mechanism^6,9,17,18^. Thus, we sought to examine the effect of Silibinin+ 5FU treated CD44+ cells on PI3K, AKT and MAPK pathways, as CD44 has been reported to have a significant role in regulating these downstream signalling pathways. In terms of the interplay between PI3K/AKT and MAPK signaling pathway, both CD44v6 siRNA transfected cells and S+ 5FU treated cells demonstrated a significant decrease in dual activation of AKT (Ser473) and ERK1/2 (Thr202/ Tyr204, Thr185/Tyr187) phosphorylation (33.44%; p < 0.0001 and 27.93%; p < 0.002 respectively) with a simultaneous increase in the number of negative cells as compared to control (21.12%; p < 0.0001 and 19.17%; p < 0.0001 respectively). Moreover, both CD44v6 siRNA transfected and Silibinin+ 5FU treated cells exhibited a significant increase in the activation of PI3K pathway (p < 0.0016 and p < 0.0115 respectively) whereas, only Silibinin+ 5FU treated cells showed an increase in the number of cells moving towards MAPK pathway (p < 0.0012; Fig. 6a,b). Collectively these results clearly depicted that targeting CD44v6 by 5FU+ Silibinin synergistic treatment reduced tumorigenic and anti-proliferative property, inhibited the migratory potential, triggered apoptosis via mitochondrial mechanism and autophagy, induced G0/G1 arrest of cell cycle and abrogates the PI3K/Akt -MAPK dual activation with near similar efficacy as siRNA mediated CD44v6 knockdown cells.

## Discussion

CD44 regulates biological and functional processes such as growth, cell invasion, migration, therapeutic-resistance and recurrence which are responsible for the progression and aggressive nature of various malignancies^19,20^. Several studies have highlighted the significance of CD44 over the last two decades; however, there was great discrepancy in these reports as all these studies fail to take into consideration that ‘CD44’ is a collection of different isoforms having distinctive functional and clinical relevance^21^. CD44v6 has been reported as functional biomarker predominantly responsible for cancer progression, initiation of metastatic process, resistance to conventional therapeutic modalities and relapse and associated with poor patient survival in colon cancer patients^9–11,22^. Therefore, the need of the hour is to develop new anti-CD44v6 specific therapeutic modalities for colon cancer patients. In this study, we predicted a comprehensive three-dimensional CD44v6 protein structure. To the best of our knowledge, this is one of the preliminary studies predicting a three-dimensional structure of CD44v6 protein having high stability and accuracy. We further screened this predicted structure against 1674 potential lead compounds comprising of FDA-approved drugs, experimental molecules and natural compounds using virtual molecular docking. Docking results suggested that Silibinin could be identified as potential antagonist that binds around the binding loop of HA with different interacting residues as well as H-bonds. Moreover, enhancement of docking results by molecular dynamic simulation analysis showed that interaction of Silibinin resulted in ligand induced conformational changes in amino acid residues located in variant v6 insertion region. Collectively, these findings suggested that Silibinin interaction with CD44v6 could potentially target this CSC by competitively inhibiting HA from binding to its binding site and induce conformational changes in CD44v6 structure which might have a plausible impact on various functions and mechanisms that govern tumor progression and metastasis in colon cancer. Previous reports suggest that Silibinin could significantly reduce tumorigenic potential by suppressing CSC subpopulation *in-vitro* in colorectal cancers and breast cancers^12,23^; however, the underlying mechanism by which Silibinin targets CD44v6 subpopulation still needs to be elucidated. Therefore, Silibinin was subjected to *in-vitro* validation in order to assess the efficacy of this potential lead compound on the molecular and functional characteristics of CD44v6 and to delineate the pathways that regulate this CSC subpopulation.

HCT116 colon cancer cell line derived CD44+ enriched population was screened in the presence of 5FU, Silibinin and 5FU+ Silibinin combination as compared to untreated cells and CD44v6 siRNA transfected cells for assessing their effect on cell viability. 5FU is a well-known anti-cancer drug and has been approved by the FDA as first line chemotherapeutic agent for the treatment of colon cancer patients^23,24^. However, these results signify that CD44+ subpopulation demonstrated no significant response to 5FU even at higher concentrations, thereby proposing that patients with presence of these CSCs might demonstrate relatively low or no response to conventional chemotherapy. Further, consistent with the previous reports^12,18,25^, we observed that Silibinin demonstrated a significant cytotoxic effect towards CD44+ subpopulation of HCT116 cell lines but at very high concentration (IC_50_ values of 250 μM). Contradictorily, we observed that 5FU-Silibinin synergistically exerted a stronger dose-dependent growth-inhibitory effect against CD44+ enriched population at considerably lower concentration (IC_50_ values of 10 μM + 50 μM respectively) as compared to Silibinin and 5FU standalone. Therefore, 5FU-Silibinin combination at the given concentration could be an effective therapeutic regimen in treating the otherwise drug resistant CD44+ subpopulation. Interestingly, 5FU+ Silibinin combination demonstrated similar anti-proliferative ability as CD44v6 siRNA transfected HCT116 cells, prompting towards its plausible role in inhibiting cell proliferation by targeting CD44v6 population.

Hence, to validate the efficacy of this combination on CD44v6 isoform, transcript and protein level expression were assessed using real time PCR and western blot analysis. Notably, 5FU+ Silibinin decreased both mRNA and protein expression of CD44v6 in HCT116 cell lines Moreover, 5FU+ Silibinin potentially decreased the expression of Nanog which infers that it can govern the vital genes in stem cell ‘niche’ that are responsible for colon CSC survival^12,26–28^. Furthermore, 5FU+ Silibinin had a significant effect in regulating expression of key regulatory signals and EMT regulatory molecules such as E-cadherin, β-catenin, AKT1 and CDKN2A that play a crucial role in regulating growth, survival, EMT progression and cell cycle mechanism in colon carcinogenesis^29^. Since, EMT is exploited by cancer cells to increase self-renewal and migration through a process that favors the emergence of CSCs^7,9^, we further evaluated the cytotoxic effect of 5FU+ Silibinin on these characteristic features of CD44+ subpopulation of HCT116. Interestingly, combinatorial effect of 5FU+ Silibinin demonstrated significant reduction in the number and size of tumor spheres formed by HCT116 derived CD44+ subpopulation compared to untreated cells. Recent report suggested that Silibinin might have a plausible role in transforming CD44+ into CD44- phenotype in colonospheres of CRC cells^12^. However, we hypothesize that 5FU effectively targets the Silibinin induced differentiated CD44- phenotype, thereby enhancing the efficacy of this combination in targeting CSC subpopulation at relatively lower concentrations. Further, the kinetic analysis of cell migration revealed that 5FU+ Silibinin co-treatment remarkably reduced the migratory potential of HCT116 derived CD44+ cells in a time- and dose-dependent manner. Collectively our results indicated an indispensable role of 5FU+ Silibinin in curbing the metastatic potential of CD44+ subpopulation by inhibiting EMT activation and migratory potential of these cells.

Further, it was inevitable to validate the synergistic effect of 5FU and Silibinin on the cell cycle progression and programmed cell death mechanism. Our results depicted that 5FU+ Silibinin co-treatment significantly induced arrest in G0/G1 phase of the cell cycle, stimulated PARP cleavage, and increased the number of apoptotic cells in CD44+ subpopulation. However, co-treatment of these drugs depicted an enhanced cell cycle arrest and apoptotic effect on the CSC population as compared to CD44v6 siRNA silenced cells. This could be attributed to the fact that Silibinin has been reported to induces growth inhibition, promotes cell cycle arrest and stimulate apoptotic cell death effect in the non-CSC cancer cells of various colon cancer cell lines such as HT-29^29,30^, LoVo^31^ and SW480 cells^32^. Collectively, these findings clearly indicated that combinatorial treatment of Silibinin and 5FU, enhanced anti-proliferative response, suppressed self-renewal and inhibited migratory potential of CSCs is attributed G0/G1 cell cycle arrest which thereby initiates programmed cell death of the CD44+ subpopulation. Subsequently, we evaluated the mechanism by which 5FU+ Silibinin trigger apoptosis in CD44+ subpopulation. Post CD44v6 siRNA and 5FU+ Silibinin treatment, HCT116 derived CD44+ cells demonstrated significant depolarization of the mitochondrial membrane potential and initiation of nuclear fragmentation. These results indicated that targeting CD44v6 either knockdown or combinatorial drug treatment had the potential to commence the programmed cell death in CSC subpopulation by pulverizing the mitochondrial integrity and activating the intrinsic apoptotic pathway. Alternatively, few reports showed a similar effect of Silibinin standalone in LoVo, SW480 and SW620 cell lines^18,33,34^. However, this inconsistency in the findings is attributed to the fact that these experiments were conducted to assess the efficacy of Silibinin on non-CSC subpopulation whereas in our study we sought to assess the efficacy of this compound in targeting CD44v6 subpopulation.

Autophagy is a protective, pro-survival response generated by the cells during stress conditions. However, if this mechanism is hyper-activated, it results in excessive degradation of essential components required for normal cellular functions leading to autophagy induced cell death^18,35^. Our results revealed that siRNA mediated knockdown of CD44v6 did not demonstrate LCI/II conversion. Conversely, drug based suppression of CD44v6 subpopulation significantly induced the LCI/II conversion with subsequent cleavage of PARP, thereby signifying autophagy mediated programmed cell death. Previous reports suggest that Silibinin, a potent anti-oxidant, has demonstrated the ability to induce autophagy induced cell death in fibrosarcoma, prostate and breast cancer cells through production of reactive oxygen species (ROS) as well as inhibiting MEK/ERK and PI3K/Akt pathways^36–39^. Therefore, we further evaluated the effect of 5FU+ Silibinin and siRNA mediated CD44v6 transfected cells on PI3K/AKT, and MAPK subfamilies in HCT116 derived CD44+ cells. Notably, both 5FU+ Silibinin synergistic treatment and CD44v6 knockdown showed a remarkable decrease in PI3K/AKT-MAPK dual activation pathways and a simultaneous increase in number of cells inactivated cells. Collectively, these results are indicative of the fact that 5FU+ Silibinin potentiates its anti-cancer activity by suppressing the dual pathway activation and inducing autophagy mediated programmed cell death. Currently, no drugs targeting CD44v6 are available in the market and hence the current approach may have considerable therapeutic implications. However, since the combinatorial treatment vaguely enhanced the activation of PI3K pathway, targeting CSC population with anti-PI3K inhibitors in the adjuvant setting may enhance therapeutic implications^40,41^.

## Methods

### Homology modeling and model validation

To model a comprehensive three-dimensional protein structure of CD44v6 **(**405 aa**)**, various sequences of CD44 isoforms were derived and used from Uniprot database. Protein structures of suitable templates were identified on basis of high sequence similarity, low e-value scores and predefined parameters by performing BLAST analysis^42^. The identified templates (PDB IDs: 1UUH, 2PF5, 4DUR, and 4MRH) and CD44v6 protein sequence were subjected to multiple template modeling approach, in order to predict an accurate three-dimensional protein structure using MODELLER 9.14^43,44^. Moreover, PROCHECK, Ramachandran plot and VERIFY-3D analysis were conducted to assess the stereo-chemical quality, overall visual quality and accuracy of the predicted CD44v6 structure^45–47^. The best modeled structure was further selected for MD Simulation in order to refine and stabilize the structure^48^.

### Molecular docking

A dataset of 1522 natural compounds, 130 FDA approved drugs and 22 investigational molecules were retrieved from NCBI PubChem database and subjected to energy minimization using YASARA Structure based Amber03 force field to obtain a stable conformation^49,50^. The homology model of CD44v6 protein was preprocessed for the stereo chemical check followed by the adding polar hydrogens. Subsequently, grid points with 0.35 Å spacing were enumerated using AutodockVina default optimization parameters^49^. Standard docking protocol was considered and the docked poses with relatively lower binding energy were selected as an optimum docked pose for further analysis. It should be noted that higher positive energies signify better binding according to YASARA energy parameters.

### Molecular dynamic simulation

For MD simulation, protein-ligand complexes were primarily prepared and processed using YASARA with AMBER14 force field. These structures were attached to a lipid bilayer membrane at their defined position to mimic the *in-vivo* condition in a cubical periodic simulation box. The grid size was adjusted to 156.2584 Å × 156.2584 Å × 156.2584 Å dimensions with other default parameters of periodic boundary conditions and energy minimized using steepest descent method. The molecular complexes were simulated for 50 ns with frame capture at every 25 ps step comprising of following parameters: (a) Harmonic restraints of molecular complexes were calibrated with force constant = 1000 kJ mol^−1^ nm^−2^; (b) Constant temperature of 298 K, pH = 7.4 and pressure of 1 bar using Berendsen thermostat and barostat respectively; (c) Particle mesh Ewald (PME) method was applied to evaluate Coulomb electrostatic interactions. Further, Root mean square deviation (RMSD), Root mean square fluctuation (RMSF) and H-bond analyses of ligand bound protein structures were conducted throughout 50 ns simulation process using YASARA, LigPlot and VMD softwares^49,51,52^.

### Cell culture and immuno-magnetic cell sorting of CD44+ subpopulation

Human colorectal carcinoma (HCT116) was obtained from National Center for Cell Science (NCCS), Pune, Maharashtra, India. HCT116 cells were cultured in RPMI-1640 medium (Gibco, Life technologies, USA) supplemented with 10% fetal bovine serum (FBS) and 1% PSN antibiotic solution (Gibco, Life technologies, USA). These cells were exposed to FITC-conjugated anti-CD44 mouse antibody (Stem Cell Technologies, USA) and were further labelled with dextran-coated magnetic nanoparticles using bispecific Tetrameric Antibody Complexes (TAC). These cells were subjected to immuno-magnetic cell separation and CD44+ cells were identified as pure cancer stem cells.

### Drug preparation and cell treatment

siRNA was synthesized using the Silencer siRNA Construction Kit (Applied Biosystems, Austin, TX), according to the manufacturer’s protocol with customized CD44v6 specific siRNA^53^ (Sequences mentioned in S1). The sorted HCT116 derived cells were transfected with 10 nm concentration of CD44v6 specific siRNA using Lipofectamine 2000 (Invitrogen, Carlsbad, CA). Scramble siRNA (Csi) was used as internal control. Simultaneously, 5FU (10 mM in DPBS), Silibinin (100 mM in DMSO) were freshly prepared and used at required concentration for various molecular and biological assays.

### Quantitative gene expression using Real Time PCR

Total cellular RNA was extracted from control, CD44v6 knockdown and Silibinin+ 5FU synergistically treated CD44+ cells from HCT116 using Trizol reagent (Invitrogen, Life Technology, USA) and reverse transcribed into cDNA using the iScript cDNA Synthesis Kit (Bio-Rad Laboratories) as per the manufacturer’s instructions. Furthermore, cDNA was amplified by Real time PCR on the AriaMx Real-time PCR System (Agilent technologies) as per manufacturer’s protocol using primer sequences (Primer sequences mentioned in S1). Each assay was normalized by using the difference in critical thresholds (CT) between target genes and 18SrRNA. The expression of mRNA of respective genes was compared with control using the values of 2^−ΔΔCT^.

### Western blotting

Protein was extracted from CD44v6 knockdown and 5FU+ Silibinin treated CD44+ HCT116 cells and the concentration was determined by BCA protein estimation kit (Sigma-Aldrich, USA). 50 μg of protein was resolved on 12% SDS-PAGE and transferred to PVDF membrane by wet electro-blotting method at 4 °C. The membrane was blocked with 5% non-fat milk in Tris buffered saline for 3 h at room temperature followed by overnight incubation with primary the primary antibodies: anti-CD44v6 (1:250), anti-PARP (1:1000), LC-3 (1:1000) and anti-β-actin (1:1000) at 4 °C. Membrane was probed with horseradish peroxidase (HRP) conjugated secondary antibodies (1:10,000). Proteins were detected using EZ-ECL kit (Clarity Western ECL substrate, BIO-RAD, USA) according to manufacturer’s instructions and signal was captured on Kodak X-Omat blue film (NEN Life Sciences, Inc., Boston, MA) in the dark^54^.

### Sphere forming assay

The immuno-magnetically-sorted CD44+ cells from HCT116 cell line were seeded in 6-well ultra-low attachment plates (Corning; New York, NY, USA) at a density of 5 × 10^3^ cells/well and cultured in low glucose RPMI, 10% fetal bovine serum and 100 U/ml Penicillin-streptomycin at 37 °C and 5% CO_2_. The medium was changed every alternative day until tumor sphere formation was observed after 2 weeks of incubation. The percentage of tumor spheres was calculated by dividing the number of spheres by the number of cells seeded per well.

### Drug cytotoxicity assay

The cytotoxic effect of 5-FU, Silibinin and 5FU+ Silibinin (Sigma Aldrich) was evaluated in HCT116 cell line using MTT (3-(4,5-Dimethylthiazol-2-yl)-2,5- Diphenyltetrazolium Bromide) assay by preparing its dilutions in media. Control and CD44v6 siRNA transfected CD44+ cells were seeded in 96-well plate at a density of 5 × 10^4^ cells/well. 5-Fluorouracil and Silibinin were added to cultured cells in different concentrations (10–1000 μM) to check their IC_50_ value at 48 h exposure. After the removal of media, 10 μl of MTT (5 mg/mL, Sigma) was added to each well and plate was incubated at 37 °C for 4 h. Then, 200 μl of DMSO was added to each well and mixed thoroughly to dissolve the formazan crystals. Absorbance was measured at 570 nm using an ELISA reader (Multiskan Spectrum Microplate Reader, Thermo Scientific). The experiments were performed in triplicates. All data were normalized to corresponding DMSO controls.

### Wound healing assay

Cells in exponential growth phase were grown in 6-well plates until they reached confluence. Using a plastic pipette tip, horizontal line across the entire diameter at the bottom of each well was scraped to induce a ‘wound’. The migration of cells was analyzed at time interval of 48 h after scraping under an inverted microscope (Eclipse TE300, Nikon) in order to compare difference in the migratory potential of CD44v6 knockdown, 5FU+ Silibinin treated and control CD44+ cells.

### Flow cytometry analysis

Control siRNA, CD44v6 specific siRNA transfected and Silibinin+ 5FU synergistically treated CD44+ cells from HCT116 cell lines were analyzed using the Muse Count and Viability kit, Annexin V and Dead Cell Assay kit, Cell Cycle Assay kit and PI3K/MAPK Dual Activation kit on Muse Cell Analyzer (Millipore, Billerica, MA, USA) according to the manufacturer’s instructions.

#### Analysis of changes in mitochondrial membrane potential (Δψ_m_)

Alteration in mitochondrial membrane potential (Δψ_m_) was analyzed using lipophilic fluorescent probe JC-1 as described earlier^55^. Briefly, 1 × 10^5^ cells were seeded on poly L-lysine coated coverslips and treated with 5FU+ Silibinin for 48 h. After incubation, residual medium was replaced with DPBS containing JC-1 dye (5 μg/ml) and incubated for 20 min in the dark. Subsequently, cells were counterstained with DAPI and were observed using fluorescence microscope (Leica, Germany). In addition, this was also confirmed by quantitative analysis by recording fluorescence at 505 nm excitation and at 527 nm (green fluorescence) and 590 nm (red fluorescence) emission respectively, using a multimode micro- plate reader (Molecular Devices, USA). Results were interpreted with ratio of red to green fluorescence representing changes in mitochondrial membrane potential (MMP).

### Analysis of nuclear morphology

Nuclear morphology was analyzed by propidium Iodide (PI) staining. Briefly, 1 × 10^5^ HCT 116 cells were seeded on poly L-lysine coated coverslips. The cells were transfected with scrambled siRNA and CD44v6 siRNA and treated with 5FU, Silibinin and 5FU+ Silibinin for 48 h. Thereafter, the cells were stained with 1 μl of Propidium Iodide (PI) for 5 min in dark at room temperature and were observed under a fluorescent microscope. More than 100 cells from three random fields were taken to examine the nuclear morphology. All the images were acquired by Image-Pro MC 6.1, (Bethesda, MD, USA) and analyzed by Image J software (NIH, USA).

### Statistical analysis

Statistical analysis was conducted by Student’s t-test using SPSS version 13. Differences were expressed as mean ± SD and data was considered statistically significant when p-values was lower than 0.05.

## Electronic supplementary material

Supp Data 1
