# Publisher Correction: Silibinin, A Natural Blend In Polytherapy Formulation For Targeting Cd44v6 Expressing Colon Cancer Stem Cells

**DOI:** 10.1038/s41598-018-36920-0

**Published:** 2018-12-17

**Authors:** Shanaya Patel, Bhargav Waghela, Kanisha Shah, Foram Vaidya, Sheefa Mirza, Saumya Patel, Chandramani Pathak, Rakesh Rawal

**Affiliations:** 1 Division of Biological & Life Sciences, School of Arts and Sciences, Ahmedabad University, Ahmedabad, Gujarat India; 2 Department of Life Sciences, School of Sciences, Gujarat University, Ahmedabad, Gujarat India; 3 Department of Cell Biology, Indian Institute of Advanced Research, Gandhinagar, Gujarat India

Correction to: *Scientific Reports* 10.1038/s41598-018-35069-0, published online 19 November 2018

The original version of this Article contained errors in the affiliation list.

Bhargav Waghela, Foram Vaidya and Chandramani Pathak were incorrectly affiliated with ‘Department of Life Sciences, School of Sciences, Gujarat University, Ahmedabad, Gujarat, India’.

In addition, Kanisha Shah, Sheefa Mirza, Saumya Patel and Rakesh Rawal were incorrectly affiliated with ‘Department of Cell Biology, Indian Institute of Advanced Research, Gandhinagar, Gujarat, India’.

The correct author affiliations are listed below:

Affiliation 1

Division of Biological & Life Sciences, School of Arts and Sciences, Ahmedabad University, Ahmedabad, Gujarat, India

Shanaya Patel

Affiliation 2

Department of Life Sciences, School of Sciences, Gujarat University, Ahmedabad, Gujarat, India

Shanaya Patel, Kanisha Shah, Sheefa Mirza, Saumya Patel & Rakesh Rawal

Affiliation 3

Department of Cell Biology, Indian Institute of Advanced Research, Gandhinagar, Gujarat, India

Bhargav Waghela, Foram Vaidya, Chandramani Pathak

