# Supplementary material for: Silibinin, A Natural Blend In Polytherapy Formulation For Targeting Cd44v6 Expressing Colon Cancer Stem Cells
Source: Sci Rep. 2018 Nov 19;8:16985. doi: 10.1038/s41598-018-35069-0 (PMC6242811; doi:10.1038/s41598-018-35069-0)
Supplement: Supplementary file 1 — Supp Data 1 [file 41598_2018_35069_MOESM1_ESM.docx]

**SILIBININ, A NATURAL BLEND IN POLYTHERAPY FORMULATION FOR TARGETING CD44v6 EXPRESSING COLON CANCER STEM CELLS**

Shanaya Patel, Bhargav Waghela, Kanisha Shah, Foram Vaidya, Sheefa Mirza, Saumya Patel, Chandramani Pathak, Rakesh Rawal

**SUPPLEMENTARY DATA:**

**S1:** Sequences of primers used for the experiments

| **Sr. No** | **Name** | **Forward Primer** | **Reverse Primer** |
| --- | --- | --- | --- |
| 1. | CD44v6 | 5′-CCAGGCAACTCCTAGTAGTACAACG-3′ | 5′-CGAATGGGAGTCTTCTTTGGGT-3′ |
| 2. | Nanog | 5′-GATTTGTGGGCCTGAAGAAA-3′ | 5′-TTGGGACTGGTGGAAGAATC-3′ |
| 3. | CDH1 | 5′-GACTCGTAACGACGTTGCAC-3′ | 5′ -GGTCAGTATCAGCCGCTTTC-3′ |
| 4. | CTNNB1 | 5′-TGGATACCTCCCAAGTCCTG-3′ | 5′-CAGGGAACATAGCAGCTCGT-3′ |
| 5. | CDKN2A | 5′-CCCAACGCACCGAATAGT-3′ | 5′-GGGGATGTCTGAGGGACCTT-3′ |
| 6. | AKT1 | 5′-GCACAAACGAGGGGAGTACAT-3′ | 5′-CCTCACGTTGGTCCACATC-3′ |
| 7. | ß Actin | 5′-TGACGTGGACATCCGCAAAG-3′ | 5′-CTGGAAGGTGGACAGCGAGG-3′ |

**S2:** Sequence of siRNA used to knockdown CD44v6 expression for the experiments:

| **Sr. No** | **Name** | **Sense** | **Antisense** |
| --- | --- | --- | --- |
| 1. | CD44v6  siRNA | AATTGTACTACTAGGAGTTGCCCTGTCTC | GCAACTCCTAGTAGTAC AATTCCTGTCTC |

**S3:** The uncropped full-length western blots of CD44v6 and β-Actin (results for Figure 4a). The chemiluminescent signals of membrane were exposed on X-ray films.


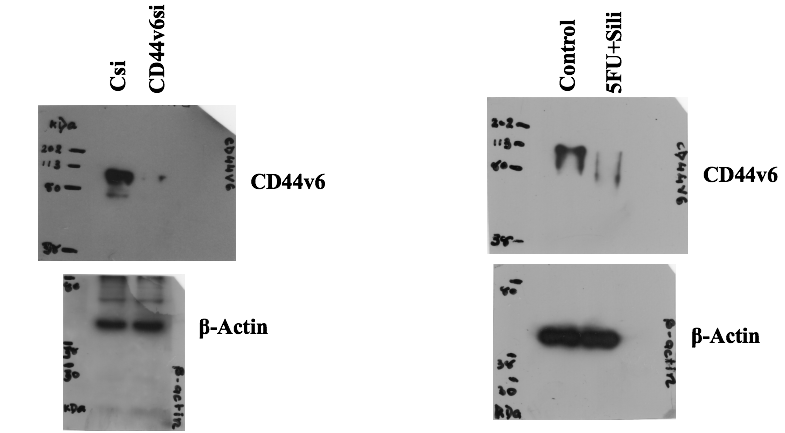


**S4:** The uncropped full-length blots of PARP, LC-3 and β-Actin (results for Figure 5c). The chemiluminescent signals of membrane were exposed on X-ray films.

**
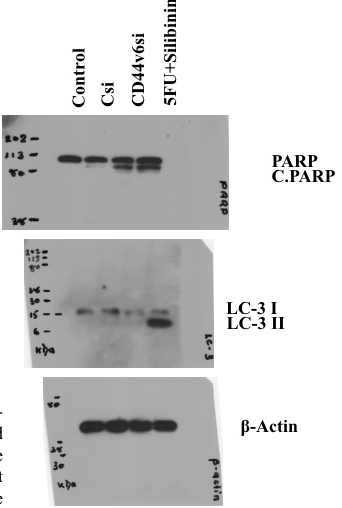
**
